# Impact of nurse-focused Baby-Friendly Hospital Initiative training program on mothers’ breastfeeding practices at hospital discharge: a quasi-experimental study

**DOI:** 10.1186/s12884-026-08993-7

**Published:** 2026-04-11

**Authors:** Abeer Salah Shaban, Samia I Hassan, Amina El-nemer

**Affiliations:** Faculty of Nursing, Mansoura University, Dakahlia Governorate, Mansoura City, Egypt

**Keywords:** Baby-Friendly Hospital Initiative, Breastfeeding practices, Nurses, Mothers, Training program, Hospital discharge, A quasi-experimental study

## Abstract

**Background:**

Maternity nurses play a key role in supporting breastfeeding and implementing the Baby-Friendly Hospital Initiative (BFHI). However, many nurses lack adequate training, and evidence on the impact of Baby-Friendly Hospital Initiative training programs on mothers’ breastfeeding practices is limited. Hence, this study aimed to evaluate the impact of Baby-Friendly Hospital Initiative training for maternity nurses on mothers’ breastfeeding practices at hospital discharge.

**Methods:**

A quasi-experimental non-equivalent two-group design was used, including a total of 94 postpartum mothers purposively assigned to the pre-intervention (*n* = 47) and post-intervention (*n* = 47) groups according to specific eligibility criteria at MansouraUniversityHospital,Egypt.Astructuredinterviewquestionnaire,whichincludedmothers’demographic characteristics and breastfeeding practices, was utilized in data collection. Mothers were interviewed individually at hospital discharge. The Baby-Friendly Hospital Initiative training program was delivered to maternity nurses working in postpartum wards and the delivery unit. The training was conducted from the beginning of November 2023 to the end of December 2023 and consisted of 10 sessions, each lasting approximately 30–45 min for a total duration of 5 to 7.5 h. Descriptive statistics, chi-square, and Monte Carlo tests were used in data analysis. Absolute risk differences and adjusted odds ratios with 95% confidence intervals were also calculated.

**Results:**

The studied mothers in the post-intervention group exhibited highly statistically significant improvements in self-reported breastfeeding practices compared to the pre-intervention group (*p* < 0.001). Major improvements were observed in assisting mothers in early initiation of breastfeeding (53.2% vs. 12.8%), exclusive breastfeeding without non-breast milk feeds (68.1% vs. 14.9%), breastfeeding on demand (70.2% vs. 25.5%), and avoidance of pacifier use (74.5% vs. 38.3%).

**Conclusions:**

This study underscores that implementing the Baby-Friendly Hospital Initiative training program for nurses was associated with improvements in mothers’ self-reported breastfeeding practices at hospital discharge and may contribute to enhancing practices aligned with the Baby-Friendly Hospital standards. These findings indicate that a continuous, comprehensive Baby-Friendly Hospital Initiative training program for all maternity staff is essential to maintain high competency and promote adherence to BFHI standards.

**Trial registration:**

ClinicalTrials.gov, NCT06824597, retrospectively registered on 31 January 2025.

**Supplementary Information:**

The online version contains supplementary material available at 10.1186/s12884-026-08993-7.

## Background

Breastfeeding is internationally regarded as the ideal source of infant feeding. As a key public health approach, it has a pivotal role in lowering morbidity and mortality rates among young children [1]. According to the World Health Organization (WHO), exclusive breastfeeding (EBF) is defined as ‘‘providing breast milk as the infant’s sole source of nutrition, either directly from the breast or through expressed milk, while permitting prescribed medications, vitamins, and minerals’’ [2].

Exclusive breastfeeding benefits the health of both infants and mothers [3]. For example, the infant receives the vital nutrition and protective antibodies from breast milk, which helps lower the risk of short and long-term health issues. These include necrotizing enterocolitis, ear infections, sudden infant death, and hospitalizations due to diarrhea and respiratory diseases [4]. Breastfeeding for a longer duration is linked to decreased rates of childhood obesity [5], type 2 diabetes [6, 7], and may offer some protection against type 1 diabetes [8, 9]. Mothers also benefit from a reduced likelihood of developing breast and ovarian cancers [10, 11].

Based on guidelines from the United Nations Children’s Fund (UNICEF), WHO, and American Academy of Pediatrics (AAP), infants should be breastfed exclusively for the first 6 months. After that, other complementary foods can be introduced while continuing to breastfeed for 2 years [12]. Notably, this modern medical guidance reflects practices that were outlined in the Holy Quran many centuries earlier [13]. Despite these international recommendations on infant feeding, the breastfeeding rates and duration remain low in many countries [14]; the global rate of EBF for infants under six months was 44% during 2015–2020 [15].

In Egypt, a descriptive study conducted in Damietta Governorate showed that most of the studied lactating mothers did not receive any prenatal breastfeeding education. Only 15.2% of them practiced exclusive breastfeeding for six months, most of them initiated breastfeeding late after birth, and 48.3% given their babies non-breastmilk feeds [16]. Furthermore, a previous descriptive study carried out in Mansoura, Egypt, found that only 14.8% of infants are exclusively breastfed at six months [17]. This means that just like in most developing countries, the exclusive breastfeeding rates are low in Egypt.

Since 1991, the WHO and UNICEF established the International BFHI as a global program to protect and support breastfeeding [18]. In 2018, WHO and UNICEF updated the BFHI guidance to strengthen implementation, focusing on institutional commitment, staff competencies, and systematic monitoring to improve breastfeeding outcomes [19]. The ‘Ten Steps to Successful Breastfeeding’ is considered the main foundation of the BFHI, which is an evidence-based and effective intervention that enhances the breastfeeding initiation, exclusivity, and duration [20]. The second step of the ‘Ten Steps to Successful Breastfeeding’ emphasizes professional training for all healthcare providers so they have the knowledge, skills, and competencies to apply evidence-based breastfeeding practices [21].

Several interconnected factors contribute to the low worldwide prevalence of breastfeeding [22]. One of these factors is the role of maternity nurses. Maternity nurses have a significant role in promoting and supporting breastfeeding worldwide. To do this effectively, they need proper breastfeeding knowledge and practical support skills [23]. However, previous literature shows that many healthcare providers lack this knowledge and these skills [24–26].

The successful breastfeeding among primiparous mothers is closely linked to the support they receive from healthcare professionals (HCPs). In developing countries, a lack of guidance from nurses and other providers during pregnancy and the immediate postpartum can create significant barriers to breastfeeding. Conversely, adequate information and support are strong predictors of positive outcomes [27]. Since primiparous mothers primarily acquire breastfeeding knowledge from maternity nurses, training these healthcare providers is critical [28]. A previous systematic review demonstrates that professional support from nurses directly enhances the initiation, duration, and maternal experience of breastfeeding [29]. Despite the global promotion of the BFHI, limited evidence exists regarding the impact of BFHI training programs for maternity staff in hospital settings, particularly in Egypt and other low- and middle-income countries. Improving nurses’ knowledge and breastfeeding support skills through structured training programs can enhance the quality of guidance provided to the postpartum mothers, thereby improving breastfeeding practices [30, 31]. Therefore, this study was commissioned to evaluate the impact of the BFHI training program for nurses on mothers’ self-reported breastfeeding practices at hospital discharge.

### Research hypothesis

Postpartum mothers who receive BFHI support from trained nurses will report better breastfeeding practices at hospital discharge than those who don’t receive such support.

## Method

### Study design

The study utilized a pre–post quasi-experimental non-equivalent groups design, comparing two non-randomized groups. A non-equivalent groups design involves comparing outcomes between groups formed by pre-existing conditions rather than through random assignment, allowing researchers to examine interventions in real-world settings [32]. Randomization was not feasible because the intervention targeted maternity nurses rather than mothers. The BFHI training program was implemented at the unit level to standardize breastfeeding practices across the postpartum departments. Its impact was subsequently evaluated on two independent cross-sectional cohorts of mothers admitted during two distinct time periods (pre- and post-intervention). As the mothers delivering in 2023 differed from those delivering in 2024, individual random allocation was neither practical nor methodologically appropriate. Therefore, the pre-intervention and post-intervention groups consisted of naturally occurring samples of mothers receiving routine care before and after the implementation of the BFHI training program.

The methodology was structured according to the standards outlined in the TREND Statement checklist [33]. The research protocol has been officially registered on ClinicalTrials.gov (NCT06824597), retrospectively registered on 31 January 2025.

### Setting

The study was conducted in the inpatient postpartum departments (wards 9, 10, 15, and 18) and the delivery unit at Mansoura University Hospital in Dakahlia Governorate, Egypt. The postpartum wards are located on the third and fourth floors, while the delivery unit is situated on the ground floor of the main building of the hospital. Each ward contains approximately 25–30 beds and includes a teaching hall. The delivery unit is made up of six rooms, one for postpartum care. These wards offer services to high-risk pregnancies and postpartum mothers across diverse cultures, both in urban and rural areas. Mansoura University Hospital represents a major tertiary teaching referral center in Dakahlia Governorate, receiving a high volume of obstetric and gynecological cases from across the Nile Delta region each year, with approximately 1,200 deliveries annually. The hospital was selected as the study setting owing to the fact that it is not an existing Baby-Friendly Hospital, which makes it the right place to apply the BFHI training program to nurses, as well as improve the breastfeeding practices of mothers according to the Baby-Friendly principles.

### Study participants

A purposive consecutive sample was used to recruit two independent groups of postpartum mothers to compare their breastfeeding practices before and after the BFHI training program. Mothers were recruited based on specific eligibility criteria: primiparous women aged 18 to 35 years, intending to breastfeed, who gave birth to a healthy singleton term baby weighing ≥ 2500 g, and were free from serious medical or obstetrical complications likely to cause mother-infant separation or decreased breastfeeding frequency (e.g., NICU admission, postpartum hemorrhage, or sepsis).

**Fig. 1:**
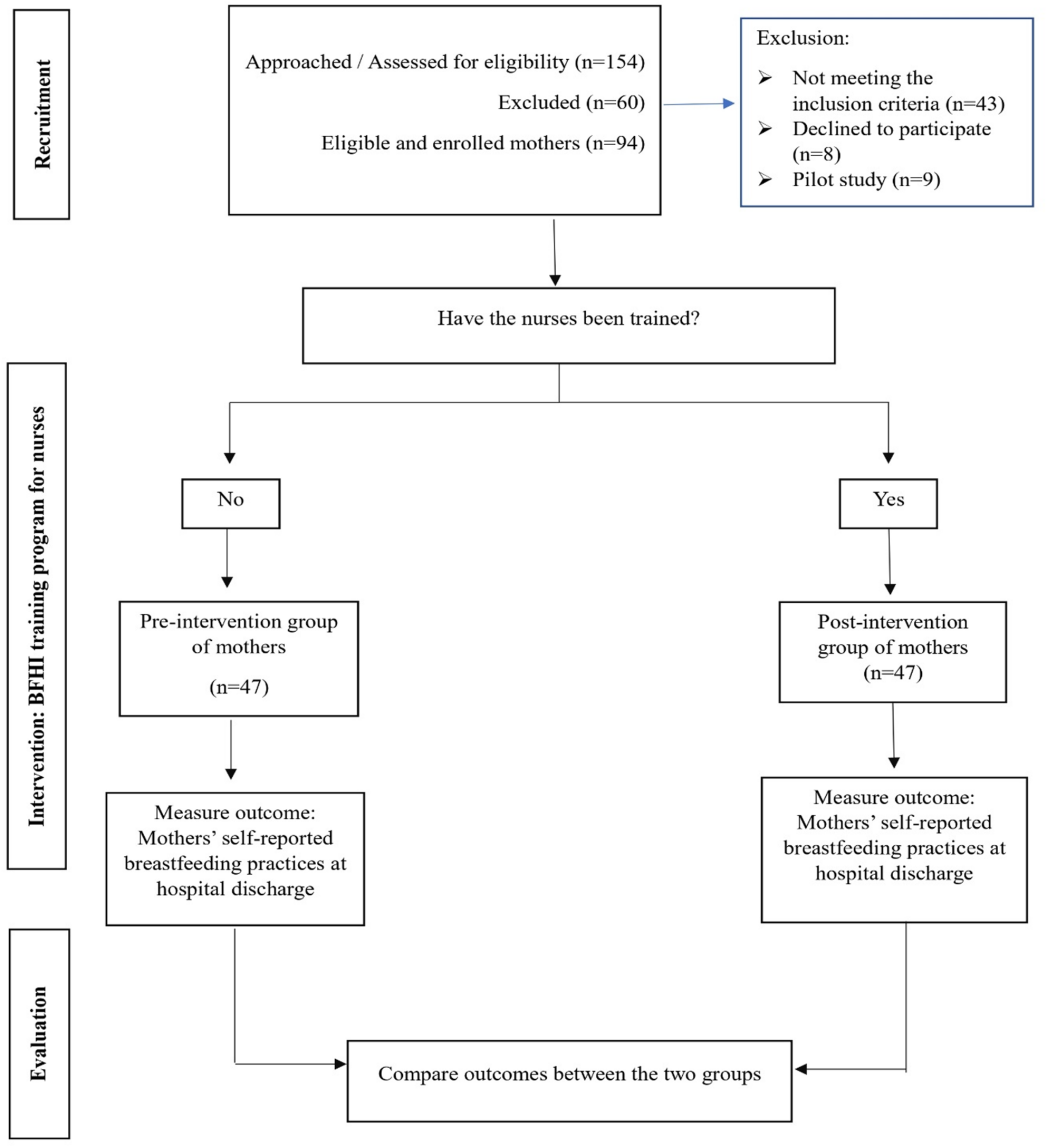
Flow diagram of study sampling

The total number of mothers assessed for eligibility, approached, declined participation, and enrolled in the study is presented in the study flow diagram (Fig. 1).

To minimize the potential confounding factors, specific eligibility criteria were applied during participant selection, and both groups were recruited from the same hospital setting under similar conditions. Furthermore, the demographic characteristics of the two groups were compared using appropriate statistical tests to ensure their baseline similarity. Additionally, no other breastfeeding training programs, policy changes, staffing changes, or institutional initiatives were introduced during the study period that could have independently influenced breastfeeding practices.

### Sample size calculation

The required sample size for this quasi-experimental study was calculated using the ClinCalc online sample size calculator for comparing two independent proportions. The calculation was based on the expected improvement in the prevalence of early initiation of breastfeeding following implementation of the Baby-Friendly Hospital Initiative (BFHI) training program. According to a previous study [34], the prevalence of early initiation of breastfeeding increased from 48% before the intervention (baseline prevalence) to 91% after the training program (expected prevalence). Using a two-sided test, a significance level (α = 0.01) and a statistical power of 95% (β = 0.05), the minimum required sample size was 74 mothers (37 in each group). To compensate for potential attrition or incomplete data during the study, 10 additional mothers were added to each group, resulting in a final sample size of 94 mothers (47 in each group).

### Data collection instrument

#### A structured interview questionnaire

It was translated into Arabic and administered by the researcher. It consisted of two parts:

Part I: Demographic and obstetric characteristics of the breastfeeding mothers, such as age, marital status, education, residence, occupation, income, and mode of delivery.

Part II: Breastfeeding mothers’ self-reported practices: It was adapted from [35] and modified to meet the study’s goal of assessing mothers’ self-reported breastfeeding practices during their hospital stay, such as timing of breastfeeding initiation, staff assistance with positioning and attaching the baby, expression of breast milk, and skin-to-skin contact. This adaptation was necessary because the original questionnaire was designed to assess breastfeeding practices during antenatal, intrapartum, and postpartum periods. For this study’s purpose, only the postpartum-related questions relevant to this aim were selected, and each question was treated as one outcome.

In the current study, the effectiveness of the training program was evaluated through mothers’ self-reported breastfeeding practices before and after the nurses’ training. Therefore, no formal practical competency assessment of nurses was conducted, as the study focused on maternal breastfeeding practices as the primary outcome.

### Validity and reliability of the instrument

The utilized instrument in this study was translated from English to Arabic following Beaton’s established guidelines to ensure linguistic equivalence and cultural appropriateness. Several systematic steps were taken to verify the accuracy and suitability of the translated instrument for the Arabic speaking population. These steps included forward translation, expert panel review, back translation, pre-testing, and cognitive interviewing. Every step was well done in order to keep the maximum accuracy and relevance in the translated questionnaire [36]. The content validity of the translated instrument was ensured through review by a panel of five experts specialized in woman’s health and midwifery nursing. The experts evaluated each item for clarity, relevance and appropriateness and their feedback was utilized to refine and improve the questionnaire items. After this validation process, the Arabic version of the instrument underwent a pilot test with 9 breastfeeding mothers, and the necessary modifications were made. The Cronbach’s α coefficient for the self-reported breastfeeding practices questionnaire was 0.874.

### Data collection

Data were collected from the pre-intervention group of mothers (served as the control group) over three months before the nurses’ BFHI training program (August to October 2023), while data from the post-intervention group (served as the case group) were obtained between June and August 2024. The principal researcher approached eligible women and invited them to participate in the study, with assistance from the head of each postpartum department and the head of the delivery unit. The principal researcher then spent approximately 5 min with each participant to explain the nature and purpose of the research and to obtain their informed written consent. Before data collection, the principal researcher received training from the research supervisors on the standardized administration of the study tools and interview procedures to ensure consistency and accuracy in data collection.

After that, the principal researcher interviewed each breastfeeding mother individually for about 10–15 min at discharge to collect their demographic characteristics and self-reported breastfeeding practices (steps 4 to 10) during their hospital stays using the breastfeeding practices questionnaire. The sole primary outcome of the study was mothers’ self-reported breastfeeding practices at hospital discharge, reflecting the implementation of BFHI steps 4–10 during the hospital stay. All study outcomes were predefined in the study protocol before data collection, and the breastfeeding-related practices were defined according to the World Health Organization (WHO) standard recommendations.

The principal researcher was also responsible for delivering the BFHI training program to the nurses and subsequently collected the study data. To minimize potential bias, standardized questionnaires and structured interview procedures were used consistently for all participants in both the pre- and post-intervention phases.

The same questionnaire was applied in the pre- and post-intervention groups. Since the data were based on mothers’ self-reported practices, there was a potential risk of recall or social desirability bias. However, interviewing the mothers at the time of hospital discharge ensured that the reported practices reflected recent experiences and minimized recall bias.

### Intervention: BFHI training program for maternity nurses

Prior to the intervention, Mansoura University Hospital did not have formal breastfeeding policies, and breastfeeding support provided by nursing staff was inconsistent and often suboptimal. Nurses’ practices regarding breastfeeding were generally poor, with limited adherence to recommended guidelines, highlighting the need for structured training to improve maternal breastfeeding practices.

The training program was conducted for 48 nurses who provide direct care to mothers and their newborns from all shifts at the aforementioned setting. It took place from the beginning of November 2023 to the end of December 2023 and included 10 sessions (both theoretical and hands-on practical), held twice a week, each lasting approximately 30–45 min for a total training duration of 5 to 7.5 h. All 48 nurses participated in the training and completed all 10 sessions, resulting in a 100% participation and completion rate. The training sessions were delivered by the principal researcher, who has experience in maternal and newborn nursing and breastfeeding support.

The training program was developed based on the UNICEF/WHO BFHI—a 20-hour course for maternity staff [37], incorporating the principles of the Ten Steps to Successful Breastfeeding. In this study, the program content was adapted and condensed to cover only steps 3–10, which are directly related to breastfeeding support practices in maternity care. Step 3, which focuses on providing breastfeeding information for pregnant women, was included in the training content because maternity nurses may also interact with pregnant women in the same units. However, this step was not included in the study’s outcomes, as the evaluation focused only on breastfeeding practices during the immediate postpartum period. Steps 1 and 2 (related to institutional policy development and staff competency monitoring) were not implemented. Therefore, the intervention primarily targeted the practice-related components of the BFHI framework relevant to maternity nurses’ clinical roles in the delivery and postpartum care. The ten steps are presented in Table 1. A detailed training plan for each session implemented during the intervention is available in supplementary file 1. To ensure intervention fidelity, all sessions were delivered according to this standardized plan, providing identical content and practical exercises to all nurses, with consistent supervision by the principal researcher.

**Table 1:** WHO ten steps to successful breastfeeding (revised 2018). Source: WHO & UNICEF (2018) [19]

**Critical management procedures**	
1. a. Comply fully with the International Code of Marketing of Breast-milk Substitutes and relevant World Health Assembly resolutions.	
1. b. Have a written infant feeding policy that is routinely communicated to staff and parents.	
1. c. Establish ongoing monitoring and data-management systems.	
2. Ensure that staff have sufficient knowledge, competence, and skills to support breastfeeding.	
**Key clinical practices**	
3. Discuss the importance and management of breastfeeding with pregnant women and their families.	
4. Facilitate immediate and uninterrupted skin-to-skin contact and support mothers to initiate breastfeeding as soon as possible after birth.	
5. Support mothers to initiate and maintain breastfeeding and manage common difficulties.	
6. Do not provide breastfed newborns any food or fluids other than breast milk, unless medically indicated.	
7. Enable mothers and their infants to remain together and to practise rooming-in 24 hours a day.	
8. Support mothers to recognize and respond to their infants' cues for feeding.	
9. Counsel mothers on the use and risks of feeding bottles, teats and pacifiers.	
10. Coordinate discharge so that parents and their infants have timely access to ongoing support and care.	

### Data analysis

The Statistical Package for the Social Sciences, version 22 (SPSS), was used to statistically analyzing data. To summarize the mothers’ demographic data and their reported breastfeeding practices, descriptive statistical methods were employed. These included frequencies and percentages for categorical data, as well as means and standard deviations for continuous variables. The Chi-square test was utilized to compare categorical variables between the pre- and post-intervention groups. Due to insufficient cell counts to meet the expected assumptions, the Monte Carlo correction was applied to obtain a more accurate p-value. The independent t-test was used to compare the mean values of continuous variables between the pre- and post-intervention groups. Estimation of effect size for significant association between the pre- and post-intervention groups using Cramer’s V and interpreted as small (0.10), medium (0.30), or large (0.50). Furthermore, absolute risk differences (ARD) with 95% confidence intervals (CI) were calculated to determine the magnitude of change between the pre- and post-intervention groups. Measures of association were evaluated using relative risk ratios (RRR) and adjusted odds ratios (AOR) with 95% confidence intervals. A p-value of < 0.05 was considered statistically significant, while a p-value of ≤ 0.001 was considered highly statistically significant.

No missing data were identified, as the questionnaire was checked for completeness at the time of data collection.

## Results

A total of 94 postpartum mothers were included in the study, with 47 assigned to the pre-intervention group and 47 to the post-intervention group. A flow diagram showing the participants’ inclusion in the study is illustrated in Fig. 1.

### Demographic and obstetric characteristics

Table 2 reveals that the mean age of the mothers in the pre-intervention and post-intervention groups was 28.23 ± 4.71 and 27.98 ± 4.91 years, respectively. All mothers in both groups were married (100%) and had attained secondary education (74.5% and 70.2%). Most mothers in both groups lived in rural areas (61.7% and 74.5%) and were housewives (68.1% and 61.7%). Additionally, the majority reported that their income was not sufficient (89.4% and 93.6%). Cesarean section was the predominant mode of delivery in both groups (72.3% and 87.2%). Among those who underwent cesarean delivery, spinal anesthesia was the most commonly used method (91.2% and 95.1%). No statistically significant demographic differences were found between the groups.

**Table 2:** Demographic and obstetric characteristics of the breastfeeding mothers

Variable	Pre-Intervention group (*n* = 47)		Post-Intervention group (*n* = 47)		Test of significant	
	No	%	No	%	χ2	*P*
Mother’s Age years						
20 to < 25 years	21	44.7	18	38.3	0.711	0.701
25 to < 30 years	17	36.2	21	44.7		
30 to 35 years	9	19.1	8	17.0		
Mean ± SD	28.23 ± 4.71		27.98 ± 4.91		t = 0.252	0.802
Marital status						
Married	47	100.0	47	100.0	1.000	1.000
Educational level						
Primary level	5	10.6	4	8.5	0.699	0.705
Secondry level	35	74.5	33	70.2		
University level	7	14.9	10	21.3		
Residence						
Rural	29	61.7	35	74.5	1.763	0.184
Urban	18	38.3	12	25.5		
Work Status						
Housewife	32	68.1	29	61.7	0.420	0.517
Employee	15	31.9	18	38.3		
Income						
Not enough	42	89.4	44	93.6	0.547	0.460
Enough	5	10.6	3	6.4		
Mode of Delivery						
Vaginal delivery	13	27.7	6	12.8	2.232	0.102
Cesarean section	34	72.3	41	87.2		
Type of anesthesia in a cesarean delivery (*n* = 34)			(*n* = 41)			
Spinal	31	91.2	39	95.1	0.465	0.495
General	3	8.8	2	4.9		

X2: Pearson Chi Square, t: independent t-test, *P* is not statistically significant if > 0.05

### Mothers’ self-reported breastfeeding practices

Table 3 demonstrates that the studied mothers in the post-intervention group showed highly statistically significant improvements in self-reported breastfeeding practices compared to the pre-intervention group(*p* < 0.001).Notable improvements were observed in mothers’assistance during the initiation of breastfeeding (53.2% vs. 12.8%, *p* < 0.001) with an absolute risk difference (ARD) of 40.4% and an adjusted odds ratio (AOR) of 7.76. Significant improvements were also observed in assistance with positioning and attaching the baby for braestfeding (57.4% vs. 21.3%, *p* < 0.001; AOR = 4.99 and 44.7% vs. 8.5%, *p* < 0.001; AOR = 8.68, respectively).

**Table 3:** Comparison of self-reported BFHI practices among mothers in the pre- and post-intervention groups

BFHI steps	Self-Reported BFHI Practices	Pre-Intervention group (n=47)		Post-Intervention group (n=47)		Test of significance				
		No	%	No	%	χ2 / Mc	P	ARD (95% CI)	RRR	AOR [95% CI]
Step 4:Early initiation of breastfeeding	Time of first holding the newborns									
	Immediately	3	6.4	6	12.8	6.128	0.190	V = 0.255		
	Within five minutes	9	19.1	16	34.0					
	Within half an hour	15	31.9	14	29.8					
	Within an hour	19	40.4	11	23.4					
	Have not held yet	1	2.1	0	0.0					
	Type of first contact									
	Skin-to-skin contact	16	34.0	23	48.9	2.147	0.143	14.9 (-4.3, 34.1)	1.44	1.86 [0.81–4.27]
	Wrapped without much skin contact	31	66.0	24	51.1					
Step 5: Maintaining breastfeeding and managing difficulties	Nurses assisted me when I first initiated breastfeeding									
	Yes	6	12.8	25	53.2	17.375	<0.001^**^^*^	40.4 (22.9, 57.9)	4.16	7.76 [2.83–21.27]
	No	41	87.2	22	46.8					
	Nurses helped me position my baby for breastfeeding before discharge.									
	Yes	10	21.3	27	57.4	12.881	<0.001^**^^*^	36.1 (17.5, 54.7)	2.70	4.99 [2.00–12.45]
	No	37	78.7	20	42.6					
	Nurses helped me attach my baby correctly for breastfeeding before discharge.									
	Yes	4	8.5	21	44.7	15.748	<0.001^**^^*^	36.2 (20.2, 52.2)	5.26	8.68 [2.66–28.35]
	No	43	91.5	26	55.3					
	Nurses showed me or provided information on how to express my milk by hand.									
	Yes	0	0.0	30	63.8	44.063	<0.001^**^^*^	63.8 (50.0, 77.6)	∞	∞
	No	47	100.0	17	36.2					
	Attempted to express milk independently									
	Yes	3	6.4	21	44.7	18.129	<0.001^**^^*^	38.3 (22.5, 54.1)	6.98	11.85 [3.20–43.87]
	No	44	93.6	26	55.3					
Step 6: Exclusive breastfeeding	Baby received non-breast milk feeds since birth									
	Yes	40	85.1	15	31.9	27.389	<0.001^**^^*^	−53.2 (−69.2, −37.2)	0.38	0.08 [0.03–0.23]
	No	7	14.9	32	68.1					
Step 7: Rooming-in	During my stay in the postpartum ward, my baby was:									
	Always with me, both day and night	36	76.6	44	93.6	5.371	0.020^*^	17.0 (3.6, 30.4)	1.22	4.07 [1.04–15.89]
	Sometimes not with me	11	23.4	3	6.4					
Step 8: Breastfeeding on-demand	Feeding frequency advice received									
	No advice given	12	25.5	7	14.9	20.956	<0.001^**^^*^	V = 0.334		
	Breastfeed on demand	12	25.5	33	70.2					
	Every hour	3	6.4	0	0.0					
	Every 1-2 hours	18	38.3	7	14.9					
	Every 2-3 hours	2	4.3	0	0.0					
Step9: No pacifiers or teats	Baby used a pacifier while in the postpartum unit									
	Yes	29	61.7	12	25.5	12.502	<0.001^**^^*^	−36.2 (−54.1, −18.3)	0.41	0.21 [0.08–0.53]
	No	18	38.3	35	74.5					
Step10: Post-discharge support	Nurses advised me where to get help for breastfeeding difficulties after returning home.									
	Yes	6	12.8	26	55.3	18.952	<0.001^**^^*^	42.5 (25.0, 60.0)	4.32	8.45 [2.99–23.87]
	No	41	87.2	21	44.7					

*χ*² Chi-square test, *Mc* Monte Carlo test, *ARD* Absolute Risk Difference (Post% − Pre%). Positive ARD = improvement, *RRR* Relative Risk Ratio (Post% ÷ Pre%), *AOR* Adjusted Odds Ratio, ∞ Infinite OR due to zero pre-intervention events, V Cramer's V (effect size: 0.10 small, 0.30 medium, 0.50 large)

(*) *P* is statistically significant if ≤ 0.05 (***) *P* is highly statistically significant if ≤ 0.001

Significant improvement were also observed in mothers’ education on hand expression of breast milk (63.8% vs. 0%), avoidance of non-breast milk feeds (68.1% vs. 14.9%), and advice on breastfeeding on-demand (70.2% vs. 25.5%). Additional BFHI practices also showed statistically significant improvements in the post-intervention group, including the practice of rooming-in around the clock, avoidance of pacifier use, and advice on breastfeeding support after discharge. However, no statistically significant differences were observed between both groups regarding skin-to-skin contact or the timing of first holding the newborn (*p* > 0.05).

## Discussion

The present study aimed to examine the impact of the Baby-Friendly Hospital Initiative training program for nurses on mothers’ self-reported breastfeeding practices. Investigating mothers’ self-reported breastfeeding practices is a critical step in incorporating maternal perspectives, which may differ from those of maternity nurses. Due to the challenges of tracking the same mothers over time, given postpartum care and hospital discharge policies, two distinct groups of mothers were compared at different time points. Consequently, the groups were non-equivalent and not randomly assigned. To minimize confounding variables and increase the likelihood that observed differences in outcomes were attributable to the intervention, the demographics of the two groups were matched as closely as possible.

These findings should be interpreted within the framework of the Baby-Friendly Hospital Initiative, focusing on the practical steps (Steps 4–10) that guide nurses’ clinical practices to support breastfeeding. The observed improvements in mothers’ self-reported breastfeeding practices suggest a potential positive association with the BFHI training program, reflecting enhanced adherence to BFHI-related practices by nursing staff. These results are consistent with a quasi-experimental study carried out in Finland, which aimed to evaluate the mothers perceptions of postpartum breastfeeding support in the hospital before and after designation to the BFHI and revealed that mothers in the post-test group perceived breastfeeding support more adherent to the BFHI standards compared to mothers in the pre-test group (*p* < 0.001) [38].

One of the most significant improvements observed in this study was the increased assistance provided by nurses to mothers during initial breastfeeding. In the post-intervention group, more than half of the mothers reported that received nurses’ support during the initial breastfeeding, assistance in positioning and attaching the baby (57.4% and 44.7%, respectively), compared to only a small proportion of the pre-intervention group reported the same support. These findings were consistent with [39], who examined the self-reported breastfeeding practices and the BFHI in Riyadh, Saudi Arabia, and found that the majority of women reported that staff encouraged and supported them to breastfeed during their hospital stay at the BFHI hospital than at the non-BFHI hospital. These improvements represent a key aspect of the BFHI step 5 and indicate increased adherence to BFHI principles by nursing staff.

Previous studies have reported positive associations between BFHI implementation and improved breastfeeding outcomes [40]. Early postpartum breastfeeding support provided by nursing staff may promote the establishment of exclusive breastfeeding practices, which is considered a step 6 of BFHI. A repeated cross-sectional study in France suggested that hospital BFHI practices were powerful predictors of exclusive breastfeeding among mothers [41]. Likewise, a Chinese study identified that the postpartum hospital stay can be the optimal time to provide breastfeeding education when there is access to professional expert advice and factual information [42].

The current finding revealed that 14.9% of mothers in the pre-intervention group were breastfed exclusively at hospital discharge, while more than two-thirds of those in the post-intervention group did so. The given finding aligns with that of Clermont et al. who carried out a study in Lebanon and noted that EBF at hospital discharge increased from 2.4% before the implementation of the program to almost half after the BFHI was implemented [43]. Furthermore, a study conducted in South Sudan supported these results [34].

Conversely, a study carried out in Saudi Arabia by Mosher et al. found that 50% of the mothers at the BFHI hospital reported that their babies received other food or drink, and 27.9% of the mothers attributed this to the nursing staff [39]. This disparity could be explained by the variation in the design of the study and the target of the intervention. Another contradictory study was conducted in Croatia during 2008–2009, which found that step 6 is the most unimplemented step of BFHI despite the Baby-Friendly status [44]. This contradiction may be due to the fact that the earlier study was conducted over a decade ago, the period when awareness, enforcement, and monitoring of the BFHI standards, particularly of the non-medically-indicated supplementation, were not as advanced as they are now.

Rooming-in is also a crucial component of BFHI (step 7), and it has been noted that some of the studies have highlighted its role in promoting both breastfeeding initiation and exclusivity [45–47]. In our study, most mothers in the post-intervention group said that their babies stayed with them throughout the day and night, unlike three-quarters of mothers in the pre-intervention group who reported the same, with significant difference (*p* = 0.020). This could be because of greater awareness and compliance to BFHI principles by the nurses. This finding is in line with a previous study in Croatia, which showed that the rooming-in practice significantly improved after the implementation of BFHI, where 98.5% of women spent the whole day and night with their babies during their hospitalization, compared to only 2% before BFHI [48]. Moreover, a more recent study in the Czech Republic concluded that adherence to step 7 of BFHI was better following the training of health care workers [49].

Notably, the current study also demonstrated that step 8 of the BFHI improved significantly upon the training program, with more than two-thirds of mothers in the post-intervention group received advice to breastfeed on-demand. This result is in agreement with a quasi-experimental study which emphasized the beneficial effect of BFHI implementation on breastfeeding practices in Hong Kong [50].

Regarding step 9, it is recommended that mothers be informed about the risks associated with using pacifiers and bottles. Several studies have demonstrated that the use of pacifiers adversely affects breastfeeding that shortening the breastfeeding duration and increasing the risk of weaning before 6 months [51, 52]. In our results, about three-quarters of the mothers didn’t use the pacifier after the intervention compared to before intervention. This finding is supported by [53], who conducted a cross-sectional study in Maryland, which demonstrated improved breastfeeding practices, especially step 9 in BFHI hospitals, than in non-BFHI hospitals.

Concerning step 10 of BFHI, over half of the post-intervention group of mothers received information from nurses about the referral places when experiencing breastfeeding difficulties after discharge, compared to only 12.8% of the pre-intervention group. This study’s finding is supported by a recent study conducted in the U.S, which revealed that adherence to step 10 is higher in BFHI hospitals than in non-BFHI hospitals [54].

The studies presented emphasize the vital importance of nurses’ role in providing breastfeeding support for the primiparous mothers and the effectiveness of the BFHI training program for improving the mothers’ self-reported breastfeeding practices, as recommended. These findings also highlight the importance of competency-based training for maternity nurses, as the combination of theoretical instruction and hands-on practical sessions may have facilitated the translation of knowledge into improved clinical support in alignment with the practice-oriented BFHI steps (Steps 4–10). However, training alone may not be sufficient to ensure sustained improvements in breastfeeding practices without supportive institutional policies and organizational commitment to BFHI implementation. Therefore, effective BFHI implementation requires a system-wide approach that extends beyond staff training to include policy development, administrative support, and integration of breastfeeding support practices into routine maternity care.

Despite the overall improvement observed in mothers’ self-reported breastfeeding practices after the training program, some practices such as the timing of initiating breastfeeding and skin to skin contact (step 4) didn’t show statistically significant variations between the pre-and post-intervention groups. This finding may be due to the high proportion of the cesarean section deliveries in both the pre- and post-intervention groups along with the widespread use of spinal anesthesia. In vaginal births, early initiation and immediate skin-to-skin contact are typically facilitated by labor and postpartum nurses who participated in the training program.

In contrast, cesarean deliveries involve operating room and recovery room staff, who were not included in the intervention. As early mother–infant contact frequently occurs within these perioperative settings, the exclusion of this group may have limited the overall impact of the training on Step 4 adherence. Furthermore, postoperative monitoring requirements and operating room routines may delay early breastfeeding initiation, particularly in settings with high cesarean rates.

The current findings were supported by the results of [54], who found that there was no significant difference in reported adherence to Step 4 between BFHI and non-BFHI hospitals. In contrast to the present findings, a comparative Indian study evaluated the breastfeeding practices before and after implementation of the BFHI program [55]. The study found a statistically significant improvement in the timing of initiating breastfeeding after the implementation of the BFHI program, with rates increasing from 39.4% to 80.6% (*p* < 0.001). This discrepancy may be attributed to most mothers in the comparative study have a higher level of education and have been exposed to BFHI for a long time, which likely enhanced their responsiveness to HCPs’ training. In contrast, the current study encountered other challenges, including an increase in caesarean section deliveries, which were likely to delay breastfeeding initiation.

The literature on the topic of the BFHI has few studies covering the impact of the program on the breastfeeding practices among mothers in Egypt. Although there are few comparable studies available, this research can be considered as a starting point for future investigations of the breastfeeding practices of mothers.

### Strengths and limitations of the study

To the best of our knowledge, this study is the first to evaluate the mothers’ self-reported breastfeeding practices after implementing the nurses’ BFHI training program in Dakahlia Governorate, Egypt. The findings indicate that changes in nurses’ breastfeeding practices exceeded observation-driven behaviors and were reflected in the quality of breastfeeding support offered to women during routine clinical care. However, some limitations in this study should be considered for future research. First, the evaluation of mothers’ breastfeeding practices was limited to the hospitalization period, which may restrict the assessment of longer-term breastfeeding outcomes. Therefore, future studies should include post-discharge follow-up assessments to evaluate the sustainability of breastfeeding practices.

Second, our research was based on mothers’ self- reported data rather than objective clinical observations of breastfeeding practices, which may have introduced recall or social desirability bias. To determine the actual effects of nurse-training programs in the field, future studies should utilize observed measures of breastfeeding practices. Third, the study employed a quasi-experimental non-equivalent two-group design in which different mothers were evaluated before and after the intervention, which may introduce a risk of selection bias. However, efforts were made to keep the two groups matched on the demographic variables, reducing this risk. Lastly, this study was conducted with a small sample of mothers at a single health care facility, therefore, the findings may not be generalized to all breastfeeding mothers in Egypt.

## Conclusions

This study underscores that implementing the BFHI training program for nurses was associated with improvements in mothers’ self-reported breastfeeding practices at hospital discharge and may contribute to enhancing practices aligned with the Baby-Friendly Hospital standards. However, step 4 of the BFHI practices remains a challenge. These results imply that a continued, comprehensive BFHI training programs for all maternity personnel, including nurses working in the operating rooms and recovery areas, are essential to maintain high competency and promote adherence to BFHI standards. Moreover, integrating the principles of the BFHI into hospital policies as part of maternity and neonatal care may help provide long-term support for breastfeeding. In this context, support from hospital administration through staff training, development of formal policies, and ongoing monitoring could facilitate the sustainability and integration of BFHI practices into the routine care.

## Supplementary Information

Supplementary Material 1.

## Data Availability

The datasets used and analyzed during the current study are not publicly available because of privacy or ethical restrictions, but are available from the corresponding author upon reasonable request.

## Abbreviations

AAP: American Academy of Pediatrics
BFHI: Baby-Friendly Hospital Initiative
HCPs: Health Care Professionals
NICU: Neonatal Intensive Care Unit
SPSS: Statistical Package for Social Sciences
UNICEF: United Nations Children’s Fund
WHO: World Health Organization
