# Supplementary material for: Impact of nurse-focused Baby-Friendly Hospital Initiative training program on mothers’ breastfeeding practices at hospital discharge: a quasi-experimental study
Source: BMC Pregnancy Childbirth. 2026 Apr 11;26:418. doi: 10.1186/s12884-026-08993-7 (PMC13085387; doi:10.1186/s12884-026-08993-7)
Supplement: Supplementary file 1 — Supplementary Material 1. [file 12884_2026_8993_MOESM1_ESM.docx]

**Training Program Plan for Maternity Nurses on Baby-Friendly Hospital Initiative (BFHI) Principles.**

**Target Audience:** Maternity Nurses working in inpatient postpartum departments and delivery unit.

**Setting:** Mansoura University Hospital

| **Session No.** | **Objectives**  At the end of these sessions, each nurse will be able to: | **Content** | **Duration** | **Teaching**  **Materials** | **Teaching Methods** |
| --- | --- | --- | --- | --- | --- |
| **Session 1.**  **BFHI: A key component of quality maternal and newborn care** | - Outline the Baby-friendly Hospital Initiative. - Discuss WHO/UNICEF Global Strategy for Infant and Young Child Feeding. - Describe the importance of exclusive and continued breastfeeding. - List the ten steps to successful breastfeeding. | - Introduction to BFHI. - WHO/UNICEF Global Strategy for Infant and Young Child Feeding. - Importance of exclusive and continued breastfeeding. - The ten steps to successful breastfeeding. | 30 minutes | PowerPoint presentation | Lecture  +  Group discussion |
| **Session 2**  **Benefits of breastfeeding** | - Identify the benefits of breastfeeding. - List the special properties of colostrum and its importance. - Describe the main differences between breast milk and artificial milk. - List the risks of artificial feeding. | - Benefits of breastfeeding. - Properties and benefits of colostrum. - Differences between breast milk and artificial milk. - Risks of artificial feeding. | 30 minutes | PowerPoint presentation | Lecture  +  Group discussion |
| **Session 3**  **Counselling skills** | - Identify key components of listening and learning in counselling. - Describe strategies for building confidence and providing support to mothers.​ - Demonstrate the appropriate use of the skills when counselling a new mother on feeding her baby. | Counselling skills:   - Listening and learning - Building confidence and giving support | 30 minutes | PowerPoint presentation | Demonstration+  Case scenario |
| **Session 4**  **How breastfeeding works** | - Identify the breast parts involved in lactation. - Explain the physiology of the lactation hormones. - Describe the breast-milk production and flow. - Differentiate between good and poor attachment of a baby at the breast - Demonstrate proper techniques for good attachment during breastfeeding | - Anatomy and physiology of lactation. - Breast-milk production and flow. - Difference between good and poor attachment of a baby at the breast - Breastfeeding attachment techniques | 30 minutes | PowerPoint presentation | Lecture  +  Demonstration |
| **Session 5**  **Impact of birth practices** | - Discuss the importance of early skin to skin contact and breastfeeding initiation. - Describe the procedure of skin-to-skin contact immediately after birth. - Identify risks of not practicing skin-to-skin contact. - Describe how health-care practices affect the initiation of breastfeeding. | - Importance of skin-to-skin contact immediately after delivery and the initiation of breastfeeding within one hour. - Procedure of skin-to-skin contact immediately after birth. - Risks of not practicing skin-to-skin contact. - Birth practices and their impact on breastfeeding. | 30 minutes | PowerPoint presentation | Demonstration |
| **Session 6**  **Breastfeeding positions** | - Explain the four key points of positioning of the baby. - Demonstrate main breastfeeding positions for the mother. - Demonstrate positions after C-section and for low-birth-weight babies. | - The four key points of positioning of the baby. - Different breastfeeding positions. | 45 minutes | Breast model  Newborn doll, video. | Demonstration+ Role play |
| **Session 7**  **Breastfeeding Challenges and Alternative Feeding Methods** | - Describe the challenges that may prevent a baby from breastfeeding effectively - List different reasons of frequent crying in newborns. - Discuss overcoming these challenges and using alternative feeding methods - Demonstrate hand expression of milk. - Explain appropriate storage methods for expressed breast milk - Describe how to help a mother who is taking medications while breastfeeding. | - Breastfeeding Challenges - Reasons of frequent crying in newborns. - Managing challenges and using alternatives (hand expression, cup feeding). - Breast milk storage - Breastfeeding and the mother’s medications | 45 minutes | - Printed booklet - Video | Group Discussion+ Demonstration |
| **Session 8**  **Milk supply challenges** | - Explain normal newborn feeding behaviour. - List the signs and symptoms of insufficient milk. - Identify the causes of milk insufficiency. - Discuss the prevention and management of milk insufficiency in newborns. - Differentiate between perceived and actual milk insufficiency | - Normal newborn feeding behaviour. - Signs and symptoms of milk insufficiency - Causes of milk insufficiency. - Prevention and management of milk insufficiency in newborns. - Difference between perceived and actual milk insufficiency. | 30 minutes | - Printed booklet  -PowerPoint presentation | Group discussion  Demonstration |
| **Session 9**  **Postnatal practices to support breastfeeding** | - Describe the importance of avoiding prelacteal feeds and unnecessary supplementation. - Outline the advantages of rooming-in. - Explain responsive feeding and and identify early feeding cues - Discuss the implications of pacifier usage | - Importance of avoiding prelacteal feeds and unnecessary supplementation. - Advantages of rooming-in. - Responsive feeding and its importance. - Early feeding cues - Implications of pacifier usage | 30 minutes | - Printed booklet. | Lecture  +  Group discussion |
| **Session 10**  **Discharge care** | - Describe preparing the mother for discharge. - List indicators of successful breastfeeding. - Recognize the warning signs related to breastfeeding after discharge. - Identify community resources to support breastfeeding. | - Discharge preparation. - Indicators of successful breastfeeding. - Warning signs related to breastfeeding that require immediate medical attention. - Community resources for supporting breastfeeding after discharge. | 30 minutes | PowerPoint presentation | Group Discussion |
